# Control of replication initiation by the Sum1/Rfm1/Hst1 histone deacetylase

**DOI:** 10.1186/1471-2199-9-100

**Published:** 2008-11-06

**Authors:** Jan M Weber, Horst Irlbacher, Ann E Ehrenhofer-Murray

**Affiliations:** 1 Zentrum für Medizinische Biotechnologie, Abteilung Genetik, Universität Duisburg-Essen, 45117 Essen, Germany; 2 Bayer Schering Pharma, 13342 Berlin, Germany

## Abstract

**Background:**

Replication initiation at origins of replication in the yeast genome takes place on chromatin as a template, raising the question how histone modifications, for instance histone acetylation, influence origin firing. Initiation requires binding of the replication initiator, the Origin Recognition Complex (ORC), to a consensus sequence within origins. In addition, other proteins bind to recognition sites in the vicinity of ORC and support initiation. In previous work, we identified Sum1 as an origin-binding protein that contributes to efficient replication initiation. Sum1 is part of the Sum1/Rfm1/Hst1 complex that represses meiotic genes during vegetative growth via histone deacetylation by the histone deacetylase (HDAC) Hst1.

**Results:**

In this study, we investigated how Sum1 affected replication initiation. We found that it functioned in initiation as a component of the Sum1/Rfm1/Hst1 complex, implying a role for histone deacetylation in origin activity. We identified several origins in the yeast genome whose activity depended on both Sum1 and Hst1. Importantly, *sum1*Δ or *hst1*Δ caused a significant increase in histone H4 lysine 5 (H4 K5) acetylation levels, but not other H4 acetylation sites, at those origins. Furthermore, mutation of lysines to glutamines in the H4 tail, which imitates the constantly acetylated state, resulted in a reduction of origin activity comparable to that in the absence of Hst1, showing that deacetylation of H4 was important for full initiation capacity of these origins.

**Conclusion:**

Taken together, our results demonstrate a role for histone deacetylation in origin activity and reveal a novel aspect of origin regulation by chromatin. These results suggest recruitment of the Sum1/Rfm1/Hst1 complex to a number of yeast origins, where Hst1 deacetylated H4 K5.

## Background

Genome duplication by DNA replication is fundamental for the propagation of genetic material in all organisms. Eukaryotic chromosomes are replicated from multiple start sites called replication origins that initiate bidirectional DNA replication. Replication initiation at these origins is best understood in the yeast *Saccharomyces cerevisiae*, where approximately 400 origins are used to replicate the DNA of the 16 chromosomes (reviewed in [1]). The ability of yeast origins to provide initiation and thus autonomous replication to plasmids has allowed the functional dissection of origin elements by measuring plasmid maintenance rates and has coined the term autonomous replicative sequence (ARS).

Plasmid maintenance studies have revealed that yeast origins have a modular structure. They all share a so-called ARS consensus sequence (ACS), which is a binding site for the origin recognition complex (ORC), the replication initiator. The six-subunit ORC complex binds to the origins in an ATP-dependent manner and, together with Cdc6 and Cdt1, recruits the MCM complex, which likely is the replicative helicase, to form the pre-initiation complex (reviewed in [1]). However, an ORC binding site alone is not sufficient to generate an origin. The ARS1 origin additionally contains three B elements that are required for full initiation [2]. The sequence closest to the ORC binding site, B1, cooperates in ORC binding and DNA unwinding [3], and B2 is required for loading of the MCM complex [4,5]. Interestingly, the B3 site is a binding site for the protein Abf1, which functions as a transcription factor elsewhere in the genome [6]. The precise function of Abf1 in initiation is not known, but may include a role in nucleosome positioning and origin site selection [7]. The involvement of transcription factors in initiation seems to be more general, because other transcription factors, Rap1 and Mcm1, have also been identified as origin binding factors that influence initiation [8,9]. Also, tethering acidic activators to origins improves initiation [10], suggesting that transcription factors have a general role in replication initiation.

Notably, individual ARS elements within the yeast genome share very little sequence conservation outside of the ACS. This observation supports the notion that yeast replication origins, in addition to ORC, bind several different auxiliary factors, among them transcription factors that aid in replication initiation, thus explaining why consensus sequences cannot easily be recognized. In this model, different subsets of origins are bound by different replication modulators that support full initiation of these origins.

In our previous work, we identified the Sum1 protein as a novel auxiliary initiation factor [11]. In contrast to the transcription factors described above, Sum1 in other contexts functions as a transcriptional repressor. It binds upstream of a number of middle sporulation genes and represses them during vegetative growth by recruiting the histone deacetylase (HDAC) Hst1 to the promoter, thus providing chromatin-mediated gene repression [12,13]. In this function, Sum1 is part of a protein complex containing Hst1 and the bridging repression factor of MSEs, Rfm1 [14].

In addition to its repressor function, Sum1 shows several links to ORC-mediated replication initiation as well as repression of the silent mating-type loci *HML *and *HMR*. The deletion of *SUM1 (sum1*Δ) is synthetically lethal with a conditional mutation in *ORC2, orc2-1*, which causes an initiation defect [11,15]. This suggests that a number of origins require Sum1 as an auxiliary factor, such that cells cannot tolerate the loss of Sum1 when ORC function is compromised. Accordingly, a number of Sum1-dependent origins have been identified [11,16]. Sum1 also shows a weak physical interaction with ORC [11]. Interestingly, a mutant version of Sum1, Sum1-1, was identified that bestows upon Sum1 an improved ability to interact with ORC [17,18]. Sum1-1 thus is aberrantly recruited to a number of origins, among them the silent mating-type locus *HMR*, where it establishes Hst1-dependent gene silencing [16-18]. Natural Sum1 binds to the *HML*-E silencer and, in cooperation with other silencer-binding factors, promotes gene silencing at *HML *[11].

Like all metabolic processes on DNA, replication initiation in eukaryotic cells must contend with the packaging of the DNA into chromatin, which generally restricts access to the DNA. Conceptually, the chromatin structure can be changed in two different ways, by the alteration of nucleosome position via chromatin remodelling, and by changes in the posttranslational modifications of the histones [19]. Origin function has been shown to depend on the chromosomal context and the positioning of nucleosomes by ORC. Nucleosomes proximal to ORC facilitate the initiation of replication, whereas covering of the origin by nucleosomes interferes with initiation [7]. Furthermore, the SWI/SNF chromatin remodelling complex in some contexts is required for full stability of plasmids with a minimal origin, and tethering of an activator to an origin can create dependence of the origin on a chromatin remodeller [20].

Replication initiation is also influenced by histone acetylation. It changes timing of origin firing in that the absence of the HDAC Rpd3 causes late origins to fire early, whereas tethering the histone acetyltransferase Gcn5 to late origins advances their time of firing [21,22]. This suggests that the deacetylated chromatin state suppresses early initiation. Furthermore, histone acetylation affects the efficiency of replication initiation at a subset of origins. The absence of the HDAC Sir2 partially suppresses the initiation defect of a *cdc6 *mutation, indicating that initiation at some origins is more efficient when the chromatin is in the acetylated state [23,24].

In this study, we asked how Sum1 exerted its function in replication initiation. We found that both *rfm1*Δ and *hst1*Δ were synthetically lethal with *orc2-1*, showing that the Sum1/Rfm1/Hst1 HDAC complex was required for Sum1's initiation function. We identified seven ARS elements whose initiation capacity depended on Sum1 and Hst1. In their absence, acetylation at lysine 5 of histone H4 was significantly increased at these origins. Also, mutation of the acetylatable lysines in the H4 tail to imitate the acetylated state caused reduced initiation of these plasmids. Taken together, our results show that Sum1 recruited the HDAC Hst1 to selected origins in the yeast genome, and that histone deacetylation by Hst1 at these origins was required for their full initiation function.

## Results

### *hst1Δ *and *rfm1Δ *were synthetically lethal with an *orc2-1 *mutation

**Figure 1:**
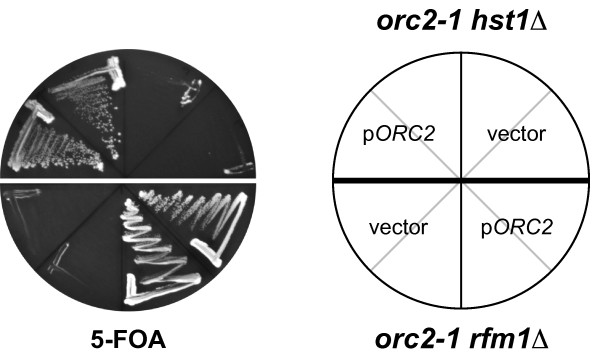
***hst1Δ *and *rfm1Δ *showed synthetic lethality with *orc2-1***. An *orc2-1 hst1Δ *(AEY3941) and an *orc2-1 rfm1Δ *(AEY3940) strain carrying p*URA3-ORC2 *(pAE1316) were tested on 5-FOA medium for their ability to lose the *ORC2 *plasmid when additionally provided with a *LEU2*-marked *ORC2 *plasmid (pAE1315) or empty *LEU2*-vector (pRS315). Strains were incubated for three days at 30°C.

Sum1 interacts with Hst1 via Rfm1 [14], and this Sum1/Rfm1/Hst1 complex represses a number of midsporulation genes. We therefore asked whether this complex was also involved in Sum1's initiation function, which is reflected in the observation that an *orc2-1 *mutation is synthetically lethal in combination with *sum1*Δ [11,15]. To this end, we investigated whether *hst1*Δ and *rfm1*Δ were also lethal with *orc2-1*. Significantly, an *orc2-1 *strain with *hst1*Δ was only able to lose an *URA3*-marked *ORC2 *plasmid on counterselective medium (5-FOA) if the strain had previously been provided with an *ORC2*-carrying plasmid with a different selection marker, but not with a vector control (Fig. 1), which was in agreement with previous work [15]. Additionally, we found that *rfm1*Δ was synthetically lethal with *orc2-1 *(Fig. 1), indicating that that the whole Sum1/Rfm1/Hst1 complex was involved in the initiation function of Sum1.

### ARS activity of selected origins depended on Sum1 and Hst1

In our previous work, we used bioinformatics analysis of genome-wide binding studies to identify regions in the *Saccharomyces cerevisiae *genome that are bound by both ORC and Sum1 and thus are good candidates for origins of replication that are regulated by Sum1 [11]. This analysis revealed eight regions that showed binding of both ORC and Sum1, and we have shown for three of these fragments that they are ARS elements and require Sum1 for full initiation capacity. To further validate these putative Sum1-regulated origins, we asked whether the genes downstream of the Sum1 binding sites were repressed by Sum1 and Hst1. To this end, we queried existing microarray data for the expression of these genes in *sum1*Δ and *hst1*Δ cells [25]. This analysis showed that six of the genes were upregulated upon deletion of *SUM1 *or *HST1 *(Table 1), indicating that the localization of Sum1 to these fragments likely recruits Hst1 to repress the neighbouring gene through histone deacetylation. In the other two cases, Sum1 may solely act as a replication factor, because it does not repress the gene next door.

**Table 1:** Gene expression change in *sum1Δ *and *hst1Δ *strains compared to wild-type

**ARS**	**Intergenic region**	**Gene**	*sum1*Δ/WT*	*hst1*Δ/WT*
433	*iYDR383C*	*NKP1*	1.0	1.0
446	*iYDR523C*	*SPS1*	21.6	12.6
447	*iYDR533C*	*HSP31*	2.3	1.6
607	*iYFR023W*	*PES4*	3.4	2.0
1013	*iYJL038C*	*YJL038C*	2.8	2.0
1109	*iYKL059C*	*MPE1*	0.8	1.0
1223	*iYLR307W*	*CDA1*	25.5	6.2
1511	*iYOL024W*	*YOL024W*	5.1	1.5

* data from [25]

**Figure 2:**
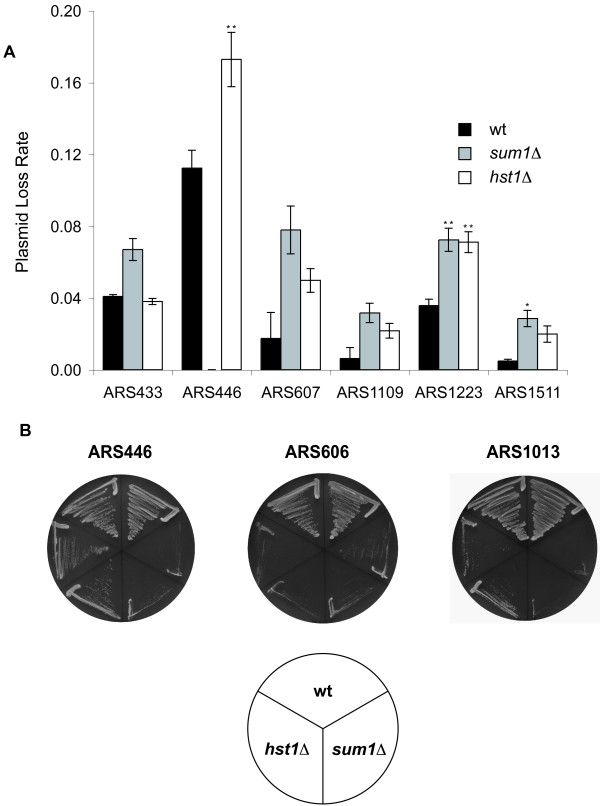
**Sum1 and Hst1 were necessary for ARS activity of selected origins**. A- Plasmid loss rates were measured in a wild-type (wt, w303), a *sum1Δ *(AEY3358) and an *hst1Δ *(AEY1499) strain. Strains carried *CEN4-URA3 *plasmids with ARS433 (pAE1240), ARS446 (pAE1250), ARS607 (pAE1242), ARS1109 (pAE1243), ARS1223 (pAE1130) or ARS1511 (pAE1135) as their sole origin. The loss rates are the average of three independent determinations. No loss rate could be determined for ARS446 in the *sum1Δ *strain. B- Primary transformants of wt, *sum1Δ *and *hst1Δ *strains carrying *CEN4-URA3 *plasmids with ARS446 (pAE1250), ARS606 (pAE1126) or AR1013 (pAE1081) were streaked on minimal plates lacking uracil and incubated for three days at 30°C. * (*P *< 0.05) and ** (*P *< 0.01) indicate statistically significant changes (student's *t *test).

We next asked whether the ARS activity of these putative origins was regulated by Sum1 and Hst1. To test this, *in vivo *plasmid maintenance of these regions was measured in wild-type, *sum1*Δ and *hst1*Δ strains. A detailed description of the ARS fragments, the presence of ACS and Sum1 binding sites and their position relative to neighbouring genes is provided in Additional file 1: Schematic representation of the ARS sequences analyzed in this study. Our earlier study had shown that ARS1013, ARS1223 and ARS1511 depended on Sum1 for full initiation [11]. Here, we found that *sum1*Δ and *hst1*Δ strains with *CEN4-URA3 *plasmids containing ARS446, ARS607, ARS1013, ARS1109, ARS1223 or ARS1511 as the sole origin displayed a significantly higher plasmid loss rate than the corresponding wild-type strain (Fig. 2A), showing that both Sum1 and Hst1 were necessary for the ARS activity of these origins. Interestingly, most origins showed a stronger dependence on Sum1 than on Hst1, reflecting the observation that many genes show stronger derepression by *sum1*Δ than by *hst1*Δ (Table 1, [25]). The effect of *sum1*Δ and *hst1*Δ on ARS activity was specific to Sum1-bound origins, because a control origin that is not bound by Sum1, ARSH4, did not show an increased plasmid loss rate [11].

In several cases, the plasmid loss was too high to measure a plasmid loss rate, because primary transformants failed to grow upon restreaking. This was the case for ARS446 in a *sum1*Δ background and for ARS1013 in *sum1*Δ and *hst1*Δ strains (Fig. 2B).

One origin, ARS433, displayed dependence on Sum1, but not Hst1 (Fig. 2A). This reflected the fact that the neighbouring gene, *NKP1*, was not repressed by Hst1 or Sum1 (Table 1) and suggested that this origin was regulated by Sum1 independently of the Sum1/Rfm1/Hst1 complex.

We further extended our analysis to the ARS606 origin that we had previously identified as a Sum1-regulated origin by searching for co-occurrences of an ACS and a Sum1 consensus-binding site [11]. ARS606 was highly unstable in *sum1*Δ as well as in *hst1*Δ strains (Fig. 2B), thus precluding the measurement of plasmid loss rates and showing that this Sum1-regulated origin also depended upon Hst1.

In contrast to other intergenic fragments, the region designated ARS447 was not capable of ARS activity, because wild-type and mutant strains transformed with ARS447 plasmids formed pinprick colonies that did not develop into viable cells after restreaking (data not shown). We tested ARS activity of a 0.5-kB as well as a 1.5-kB fragment comprising the putative ORC and Sum1-binding region, but both failed to support autonomous replication. This was in agreement with the fact that this region is designated ARS447 by [26], but not in the *Saccharomyces *Genome Database (SGD) (see Additional file 1: Schematic representation of the ARS sequences analyzed in this study).

In summary, these data showed that seven origins that were bound by Sum1, required both Sum1 and Hst1 for full initiation activity, suggesting that Sum1 recruited the Sum1/Rfm1/Hst1 complex to these origins, and that histone deacetylation by Hst1 contributed to initiation function.

### *sum1Δ *and *hst1Δ *caused increased histone H4 aceylation at selected origins of replication

**Figure 3:**
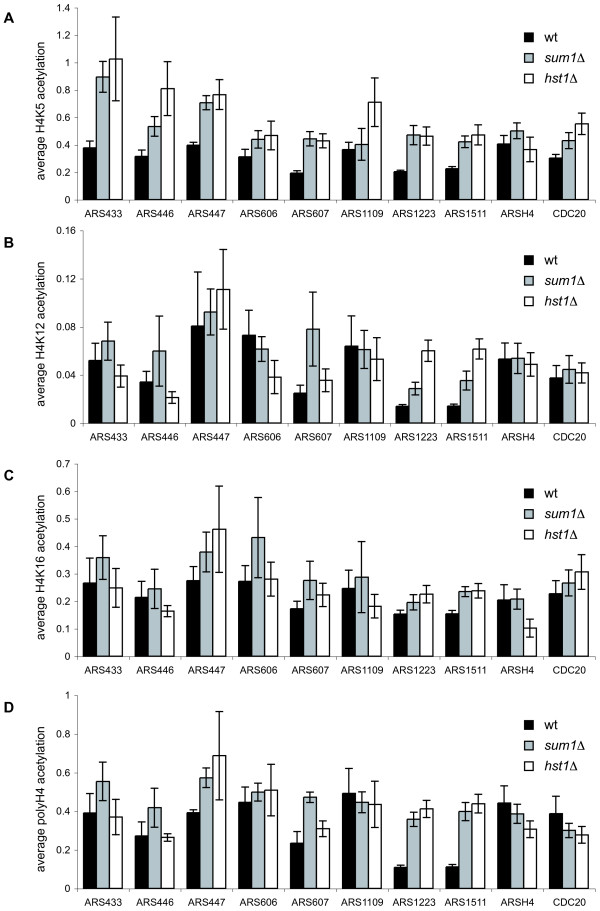
***sum1Δ *and *hst1Δ *caused increased acetylation of H4 K5 at selected origins of replication**. The amount of DNA from immunoprecipitated wild-type (wt, AEY2), *sum1Δ *(AEY3358) and *hst1Δ *(AEY1499) strains with anti-acetyl-histone H4 antibodies is shown relative to the input DNA. Quantitative qPCR was performed for eight selected origins and two controls (ARSH4 and CDC20). Error bars represent the average of six samples from two independent experiments. A- anti-acetyl-histone H4 K5, B- anti-acetyl-histone H4 K12, C- anti-acetyl-histone H4 K16, D- anti-acetyl-histone H4.

The dependence of origin function on the HDAC Hst1 suggested that histone deacetylation was necessary for efficient initiation activity. We therefore determined whether acetylation levels at these origins increased in the absence of Sum1 or Hst1. *hst1*Δ has previously been shown to moderately increase H3 and H4 acetylation [27], but Hst1 specificity so far has not been determined. For this purpose we performed chromatin immunoprecipitations (ChIP) with antibodies against different histone H4 acetyl-lysine residues. Importantly, we found that acetylation of H4 K5 was significantly increased at most ARS in the absence of Sum1 and of Hst1 (Fig. 3A and Additional file 2: Significance levels for increased H4 acetylation in *sum1*Δ and *hst1*Δ strains). These results indicated that H4 K5 was a target for deacetylation by Hst1. In contrast, H4 K5 acetylation was not increased at a control region not bound by Sum1, ARSH4 (Fig. 3A). *CDC20 *served as an additional control region that showed an increase in acetylation with the H4 K5 antibody, which was only significant in one of the two experiments (Additional file 2: Significance levels for increased H4 acetylation in *sum1*Δ and *hst1*Δ strains), but not with the other antibodies (Fig. 3).

Furthermore, this observation suggested that Hst1 directly affected replication initiation at these origins by deacetylating histone H4 K5. There were three exceptions to this scenario. ARS433 plasmid maintenance was independent of Hst1, but ARS433 showed an Hst1-dependent increase in H4 K5 acetylation. Furthermore, Sum1 was required for full ARS activity of ARS606 and ARS1109, but apparently did not affect their acetylation state (Fig. 3A). Thus, there seem to be scenarios where the relationship between Sum1, histone deacetylation and initiation is more complex. In contrast to H4 K5, H4 K12 acetylation was not significantly increased at Sum1- and Hst1-dependent ARS elements. Whereas ARS1223 and ARS1511 showed a higher amount of H4 K12 acetylation in *hst1*Δ (*P *< 0.05 Additional file 2: Significance levels for increased H4 acetylation in *sum1*Δ and *hst1*Δ) cells, the effect for ARS607 was only significant in the *sum1*Δ strain (*P *< 0.05 Additional file 2: Significance levels for increased H4 acetylation in *sum1*Δ and *hst1*Δ strains). The other origins showed no increase in acetylation (Fig. 3B). This suggested that H4 K12 was not a major target for deacetylation by Hst1, and that this site did not contribute to the regulation of initiation.

Similarly, our analysis indicated that H4 K16 was not a general target of deacetylation by Hst1. Only ARS1223 and ARS1511 showed a significant higher H4 K16 acetylation level in both *sum1*Δ and *hst1*Δ yeast strains (Fig. 3C, Additional file 2: Significance levels for increased H4 acetylation in *sum1*Δ and *hst1*Δ strains).

ChIP analysis with a poly-acetyl-H4 antibody showed overall increases in the acetylation levels at these origins in *sum1*Δ and *hst1*Δ strains. Whereas ARS1223 and ARS1511 showed a several fold higher acetylation, the effect was weaker for ARS447 and ARS607 and only significant for the *sum1*Δ strain. ARS433, ARS446 showed no significant increase in H4 acetylation, and ARS606 and ARS1109 had the same state of acetylation in all three strains (Fig. 3D). This was consistent with the notion that H4 K5 was the main target of the histone deacetylase Hst1, whereas other histone H4 lysine residues were minor or no targets of Hst1.

In summary, this analysis suggested that Sum1 regulated initiation at selected origins by recruiting Hst1 to these regions.

### Changes in H4 acetylation caused defects in plasmid stability

**Figure 4:**
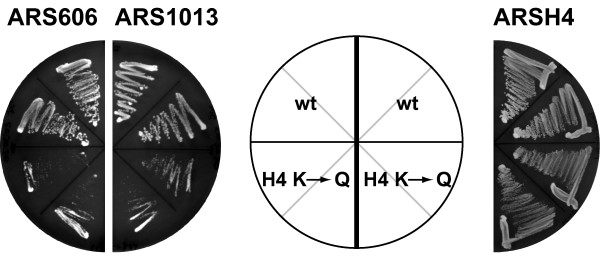
**Mutation of lysine to glutamine in the N-terminal tail of histone H4 caused a similar initiation defect as *sum1Δ *and *hst1Δ***. A wild-type (AEY3973) and a mutant strain (AEY3974) with K5, 8, 12 and 16 of histone H4 mutated to glutamine carrying *CEN4-URA3 *plasmids with ARS606 (pAE1126), ARS1013 (pAE1081) or ARSH4 (pRS316) were streaked on minimal medium lacking uracil and incubated for three days at 30°C.

The previous experiments raised the question whether increases in histone acetylation at origins by *sum1*Δ or *hst1*Δ were responsible for the loss of origin activity. To test this, we asked whether the activity at these origins was decreased in a strain in which the acetylatable lysine residues of the H4 N-terminus (K5, 8, 12 and 16) were mutated to glutamine. These mutations mimic a constant acetylation state of the respective histone H4 residue as is the case in the absence of the HDAC Hst1. We then analyzed the effect of this mutation on plasmid stability of the two plasmids that were most strongly affected by Sum1 and Hst1, ARS606 and ARS1013. Significantly, strains with ARS606 or ARS1013 plasmids showed a reduced growth rate in an H4 mutant strain as compared to wild-type (Fig. 4), indicating a loss of plasmid stability. In contrast, ARSH4, whose acetylation level did not alter in the absence of Hst1 or Sum1, did not show a reduced growth rate in the H4 mutant strain (Fig. 4). It has previously been reported that a simultaneous mutation of histone H4 K5, 8, 12, 16 to glutamine lengthens the cell cycle [28]. However, the observation that we did not detect a difference in the growth ability of mutant cells compared to wild type with the control plasmid (ARSH4) indicated that the growth differences with ARS606 and ARS1013 were specific to these origins and not due to a generalized growth defect of the H4 mutation. In summary, this suggested that increases in the acetylation level in *sum1*Δ and *hst1*Δ cells were responsible for the reduced origin activity of these ARS elements.

## Discussion

Replication initiation in eukaryotic cells takes place on the chromatin template, which raises the question how the modification state of histones influences initiation. In this study, we found that the DNA binding factor Sum1 recruited the histone deacetylase Hst1 within the Sum1/Rfm1/Hst1 complex to selected yeast origins to deacetylate histone H4. We found the most prominent effect for deacetylation of H4 K5, whereas other H4 acetylation sites were only affected at a minority of the origins.

How is histone deacetylation beneficial for initiation? The deacetylation of H4 K5 may help to stabilize the position of nucleosomes around the origin, for instance by altering DNA-histone contacts in the nucleosome, which has been shown to be important for initiation efficiency [7]. Alternatively, a particular deacetylated histone residue may recruit a histone-binding protein (complex) that recognizes H4 K5 in the deacetylated state and that has a positive effect on initiation. For instance, the acetylation may serve to recruit a chromatin remodeller that places the surrounding nucleosomes at a position that is conducive to initiation, which is in line with the observation of a role for chromatin remodellers in initiation [20]. It is also possible that Sum1/Rfm1/Hst1 indirectly affects initiation by altering the expression of genes encoding replication proteins.

It is interesting to note that we found histone deacetylation by Hst1 to be beneficial for initiation, whereas the HDAC Sir2 seems to negatively regulate initiation [23,24]. Pappas et al. identified several origins whose plasmid maintenance capacity improved upon deletion of *SIR2*, while we found a decreased maintenance rate for a different set of origins in the absence of Hst1. Also, both *hst1*Δ and *sir2*Δ affected the survival of mutations in genes encoding replication factors, but *hst1*Δ reduced their viability (Fig. 1), whereas *sir2*Δ enhanced it [23,24]. This indicates that the two HDACs both have a global effect on replication initiation, but that they influence initiation in opposite directions. This disparity may be explained by the different substrate specificities of the two enzymes. We show here that Hst1 mainly deacetylates H4 K5, while the main target of Sir2 is H4 K16 [29]. The difference in specificity may lead to the recruitment of separate sets of regulatory factors that have different effects on nucleosome positioning and initiation. Thus, the effect of histone acetylation on efficiency of a particular origin seems to be highly dependent on the chromatin context of the origin. This is comparable to the effect of chromatin remodellers on transcription, where remodelling at one promoter can lead to the exposure of a transcription factor binding site, and thus to enhanced transcription, whereas remodelling at another promoter may lead to the occlusion of a binding site, and hence reduced expression of that gene [30].

It is also possible that the Sum1/Rfm1/Hst1 complex affects the time during S phase when an origin becomes active, much like increased histone acetylation at an origin by targeted Gcn5 or by the absence of Rpd3 advances initiation [21,22]. In this respect, *sum1*Δ and *hst1*Δ may delay the firing of many origins, such that the origins are inactivated by replication forks emanating from earlier origins and thus decreased in their firing efficiency, leading to synthetic lethality in *orc2-1 *cells.

A further possibility is that the Sum1 complex influences initiation via an effect on transcription of the neighbouring gene, since transcription has previously been shown to influence origin firing [31].

Although Hst1 is an HDAC, it is also conceivable that deacetylation of a non-histone target, for instance a pre-RC component or other regulator of initiation, has an impact on initiation. However, our observation that mutation of the acetylatable lysine residues in H4 caused a similar effect on initiation as the deletion of *HST1 *lends support to the notion that Hst1 affected initiation through histone deacetylation.

The fact that the Sum1/Rfm1/Hst1 complex becomes essential when ORC function is compromised by the *orc2-1 *mutation implies that a considerable number of origins in the yeast genome require this complex for initiation. One might then postulate that replication of the genome is not efficient enough in the absence of the Sum1 complex to support viability when initiation is reduced by an *orc *mutation. However, in our analysis we identified only seven origins as being regulated by this mechanism, a number that seems insufficient to explain the synthetic lethal effect. One possibility is that more Sum1-regulated origins exist that we have not identified in our analysis. Perhaps a re-analysis of the Sum1 localization data yields new Sum1 binding sites, as was the case for the re-evaluation of estrogen receptor binding data [32].

Alternatively, the Sum1 complex may have additional functions that affect a second pathway parallel to ORC function. Next to its role in replication initiation, ORC also has a role in sister chromatid cohesion in that it mediates the interaction of sister chromatids in a pathway parallel to the interaction mediated by cohesin complexes [15,33]. Therefore, one explanation for the lethality between *sum1*Δ/*rfm1*Δ/*hst1*Δ and *orc2-1 *is that it reflects an additional role for the Sum1/Rfm1/Hst1 complex in sister chromatid cohesion. However, since *sum1*Δ cells show no defect in sister chromatid cohesion [33], this Sum1-mediated cohesion pathway may act as a back-up in cells where ORC-mediated cohesion is impaired.

## Conclusion

In summary, we have identified a role for histone deacetylation by the Sum1/Rfm1/Hst1 complex in enhancing the efficiency of replication initiation at a subset of origins. As a generalized model, our work implies that DNA binding proteins that bind close to an ORC binding site, aid in replication initiation by recruiting chromatin-modifying activities, in this case a histone deacetylase, to the origin. We postulate that deacetylation of H4 K5 helps to position nucleosomes near the origin in a location favourable to replication initiation. Given that transcription factors are also involved in initiation in metazoans [34], it will be interesting to see how they affect chromatin modification states at origins in multicellular organisms.

## Methods

### Strains and plasmids

The yeast strains and plasmids used in this study are listed in Tables 2 and 3, respectively. All yeast and *E. coli *manipulations were carried out according to standard protocols [35]. The *hst1*Δ and *rfm1*Δ gene disruptions were performed using the *KanMX *cassette according to the guidelines of EUROFAN [36] and verified by PCR.

**Table 2:** *Saccharomyces cerevisiae *strains used in this study

**Strain**	**Genotype**
AEY2	*MAT***a ***can1-100 ade2-1 his3-11,15 leu2-3,112 trp1-1 ura3-1 *(W303-1A)
AEY1499	AEY2 *hst1Δ::KanMX*
AEY3358	AEY2 *sum1Δ::HisMX*
AEY3973	AEY2, but *hht1-hhf1Δ::LEU2 hht2-hhf2Δ::HIS3, lys2Δ::hisG *+ p*CEN4- TRP1 HHF1-HHT1*
AEY3974	AEY2, but *hht1-hhf1Δ ::LEU2 hht2-hhf2Δ::HIS3, lys2Δ::hisG *+ p*CEN4- TRP1 hhf1-10(H4K5,8,12,16Q) HHT1*
AEY3940	AEY2, but *MAT***α ***orc2-1, rfm1Δ::KanMX *+ pRS316-*ORC2*
AEY4140	AEY2, but *orc2-1, hst1Δ ::KanMX *+ pRS315-*ORC2 *+ pRS316-*ORC2*

**Table 3:** Plasmids used in this study

**Plasmid**	**Description**	Source*
pRS315	*CEN6-URA3 *+ ARSH4	[38]
pAE1076	*CEN4-URA3 *+ ARS1012	O. Aparicio
pAE1081	*CEN4-URA3 *+ ARS1013-3	O. Aparicio
pAE1126	*CEN4-URA3 *+ ARS606	
pAE1130	*CEN4-URA3 *+ ARS1223	
pAE1135	*CEN4-URA3 *+ ARS1511	
pAE1240	*CEN4-URA3 *+ ARS433	
pAE1242	*CEN4-URA3 *+ ARS607	
pAE1243	*CEN4-URA3 *+ ARS1109	
pAE1250	*CEN4-URA3 *+ ARS446	
pAE1252	*CEN4-URA3 *+ ARS447	
pAE1315	pRS315-*ORC2*	
pAE1316	pRS316-*ORC2*	

* Unless indicated otherwise, plasmids were from the laboratory collection or constructed during the course of this study.

### Plasmid constructions

ARS fragments (length approx. 500 bp; ARS446 and ARS447 1500 bp) containing ARS and Sum1 consensus sequences (ACS: WTTTAYRTTTW; SUM1: DSYGWCAYWDW) were amplified via PCR from genomic DNA of wild-type cells and subcloned into the pGEM-T Easy vector (Promega) (for details, see Additional file 1: Schematic representation of the ARS sequences analyzed in this study). The vector pAE1076, which contains ARS1012, was used as a backbone for construction of the *CEN4-URA3*-ARS plasmids. It was digested with *EcoR*I and *Hind*III to release the ARS1012 fragment, and the new ARS fragments with compatible overhangs were ligated into the vector. The final constructs were verified by sequence analysis. Primer sequences are available from the authors upon request.

### Plasmid maintenance assay

Plasmid loss rates were determined for wt (AEY2), *sum1Δ::HisMX *(AEY3358) and *hst1Δ::KanMX *(AEY1499) carrying *CEN-URA3 *plasmids containing the different ARS elements as follows. Yeast transformants were grown to stationary phase in liquid minimal medium lacking uracil, and cultures were used to inoculate YPD supplemented with adenine, histidine, leucine, lysine, tryptophan and uracil. Cells were grown for at least 12 doublings at 30°C with shaking. Before and after the incubation, equal amounts of the cultures were plated on minimal medium with or without uracil. The plasmid loss rate (L) was determined by measuring the fraction of cells containing the plasmid before (F_i_) and after (F_f_) incubation in full medium as 1–10^x ^with x = [log(F_i_) - log(F_f_)]/number of doubling times [37]. The loss rate is therefore equivalent to the fraction of daughter cells that have received no plasmid during the previous cell division.

### Antibodies

The following antibodies were used in this study (rabbit antiserum, upstate): Anti-acetyl-Histone H4 (Lys5 Catalog # 07-327 Lot # 30417); Anti-acetyl-Histone H4 (Lys12 Catalog # 07-595 Lot # 28885); Anti-acetyl-Histone H4 (Lys16 Catalog # 07-329 Lot # 32214); Anti-acetyl-Histone H4 (polyclonal antiserum Catalog # 06-866 Lot # 20667).

### Chromatin immunoprecipitation (ChIP)

100 ml of yeast cells were grown to an OD_600 _of 1 and cross-linked with 1% formaldehyde for 30 minutes with shaking at room temperature. Cells were harvested by centrifugation (3 minutes, 4,000 × g) and washed twice in 1× TBS. Pelleted cells were resuspended in 100 μl ice-cold lysis buffer (50 mM HEPES (pH7.5), 1 mM EDTA, 140 mM NaCl, 1% (v/v) Triton X-100 and 0.1% sodium deoxycholate) containing protease inhibitors and disrupted with glass beads. The cell supernatant was sonicated four times for ten seconds with 200 ms impulses, centrifuged, and the protein concentration was adjusted with lysis buffer. One aliquot was taken as an input control for the quantitative real-time PCR. Aliquots were precleared with protein G-agarose (Sigma) for 2 h at 4°C and incubated over night with 3 μl antibody. After incubation, the lysates were treated with protein G-agarose-beads. The immunoprecipitates were washed with 1 ml of the following buffers (ice-cold): 1. low salt solution (0.1% (v/v) SDS, 1% (v/v) Triton X-100, 2 mM EDTA, 20 mM Tris (pH 8.1) 150 mM NaCl), 2. high salt solution (0.1% (v/v) SDS, 1% Triton (v/v) X-100, 2 mM EDTA, 20 mM Tris (pH 8.1) 500 mM NaCl), 3. LiCl buffer (0.25 M LiCl, 1% (v/v) Nonidet P-40, 1% (w/v) sodium deoxycholate, 1 mM EDTA, 10 mM Tris pH 8.1), twice 1× TE. The samples and the input DNA were subsequently treated with elution buffer (1% (v/v) SDS, 0.1 M NaHCO_3_), incubated over night at 65°C to reverse cross-linking and incubated 1 h with proteinase K (Roche). The DNA was extracted with chloroform-phenol-isoamylalcohol and precipitated with ethanol.

### Quantitative real-time PCR

The ChIP and the input samples were used in different dilutions as templates for the PCR with SYBR Green RealMasterMix (Eppendorf). The reference dilutions were used to generate a standard curve that was taken to determine the DNA amount of the ChIP samples. Fragments of 211 to 356 bp in size were amplified (see Additional file 1: Schematic representation of the ARS sequences analyzed in this study). The real-time PCR setup was as follows: An initial denaturation step at 94°C for 2 minutes, followed by 45 cycles of denaturation at 94°C for 15 seconds, annealing at 56°C for 30 seconds and elongation at 68°C for 40 seconds. After cooling to 40°C for 2 minutes and then 1 minute at 50°C, the temperature was raised every 5 seconds in 1°C intervals up to 95°C. The template amount of the immunoprecipitated samples was measured as the mean value of three dilutions relative to the computer-calculated standard curve of the input reference. Evaluation of the data comprised two independent ChIPs with standard error of the mean (six data values). Separate *P*-values were calculated for changes in acetylation levels of the mutant strains in comparison to the wild-type from the three samples of each independent ChIP (see Additional file 2: Significance levels for increased H4 acetylation in *sum1*Δ and *hst1*Δ strains).

## Authors' contributions

JMW performed all experiments and drafted the manuscript. HI provided data for the plasmid loss assay and carried out initial experiments on Sum1. AEE-M conceived and coordinated the work. JMW and AEE-M wrote, and all authors approved the final manuscript.

## Supplementary Material

Additional file 1 **Schematic representation of the ARS sequences analyzed in this study**. Genomic fragments used for plasmid loss assays are indicated by dashed, vertical lines. Black arrows represent the positions of the oligonucleotides used for quantitative real-time PCR for ChIP analysis. ARS sequences annotated in the *Saccharomyces *genome database (SGD) are given by the marked boxes (ARS447, which is not annotated in SGD, is marked in brackets). The white and black rectangles show the position of the ARS and Sum1 consensus sequences with at least 10 of 11 matches on the Watson or Crick strand. Neighbouring genes are represented by (open or closed) boxes with the respective ORF or gene name. Click here for file

Additional file 2 **Significance levels for increased H4 acetylation in *sum1Δ *and *hst1Δ *strains**. The amount of DNA from immunoprecipitated wild-type (wt, AEY2), *sum1Δ *(AEY3358) and *hst1Δ *(AEY1499) strains with anti-acetyl-histone H4 antibodies is shown relative to the input DNA. Quantitative real-time PCR was performed for eight selected origins and two controls (ARSH4 and CDC20). Diagrams show the results for the two independent experiments. Error bars represent the average of three samples each. * (*P *< 0.05), ** (*P *< 0.01) and *** (*P *< 0.001) indicate statistically significant changes (student's *t *test). A- anti-acetyl-histone H4 K5, B- anti-acetyl-histone H4 K12, C- anti-acetyl-histone H4 K16, D- anti-acetyl-histone H4. Click here for file
