# Supplementary figures and images for: Control of replication initiation by the Sum1/Rfm1/Hst1 histone deacetylase
Source: BMC Mol Biol. 2008 Nov 6;9:100. doi: 10.1186/1471-2199-9-100 (PMC2585588; doi:10.1186/1471-2199-9-100)

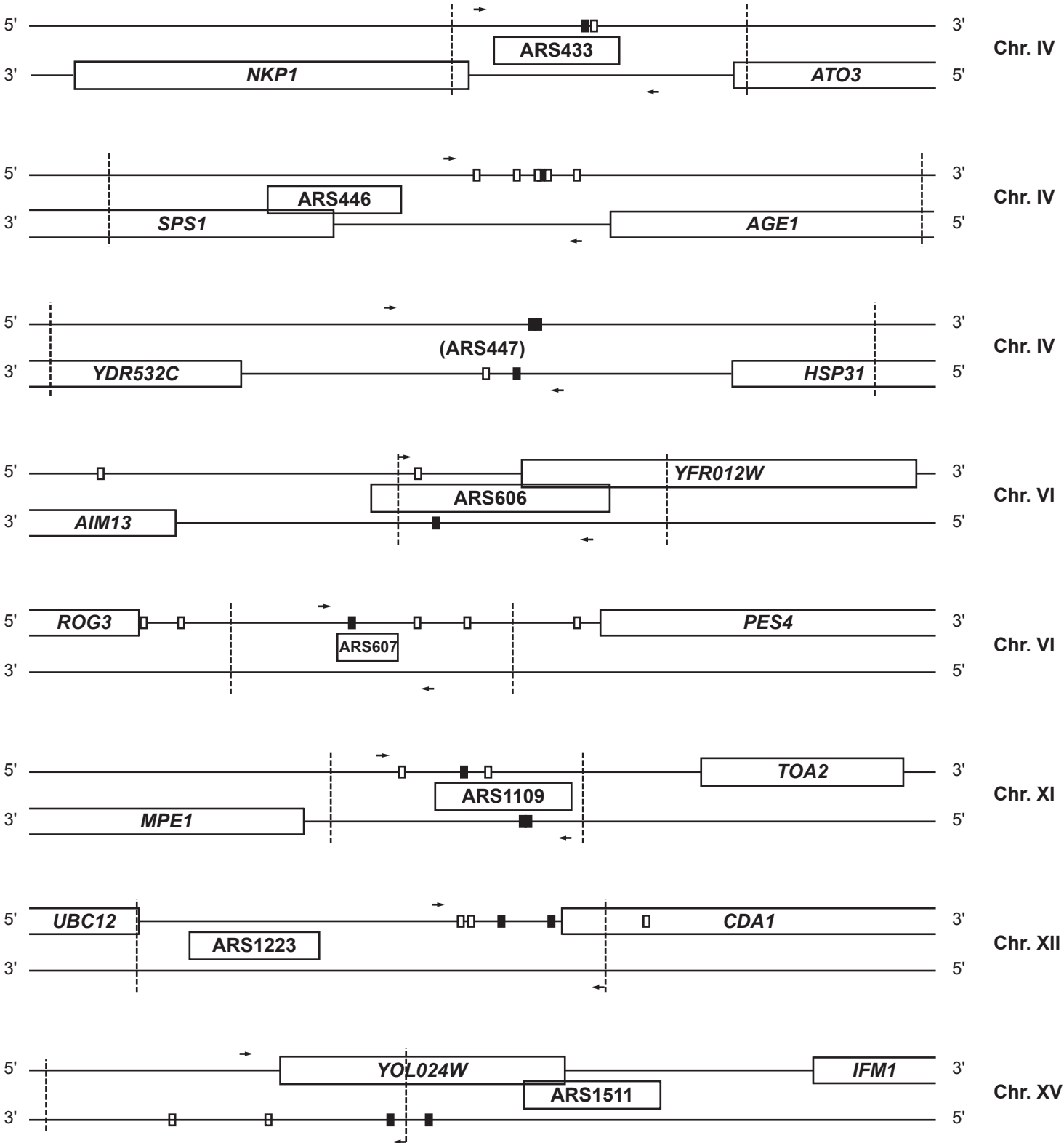

Supplement: Additional file 1 — Schematic representation of the ARS sequences analyzed in this study. Genomic fragments used for plasmid loss assays are indicated by dashed, vertical lines. Black arrows represent the positions of the oligonucleotides used for quantitative real-time PCR for ChIP analysis. ARS sequences annotated in the Saccharomyces genome database (SGD) are given by the marked boxes (ARS447, which is not annotated in SGD, is marked in brackets). The white and black rectangles show the position of the ARS and Sum1 consensus sequences with at least 10 of 11 matches on the Watson or Crick strand. Neighbouring genes are represented by (open or closed) boxes with the respective ORF or gene name. [file 1471-2199-9-100-S1.pdf]

# Experiment I

**A**

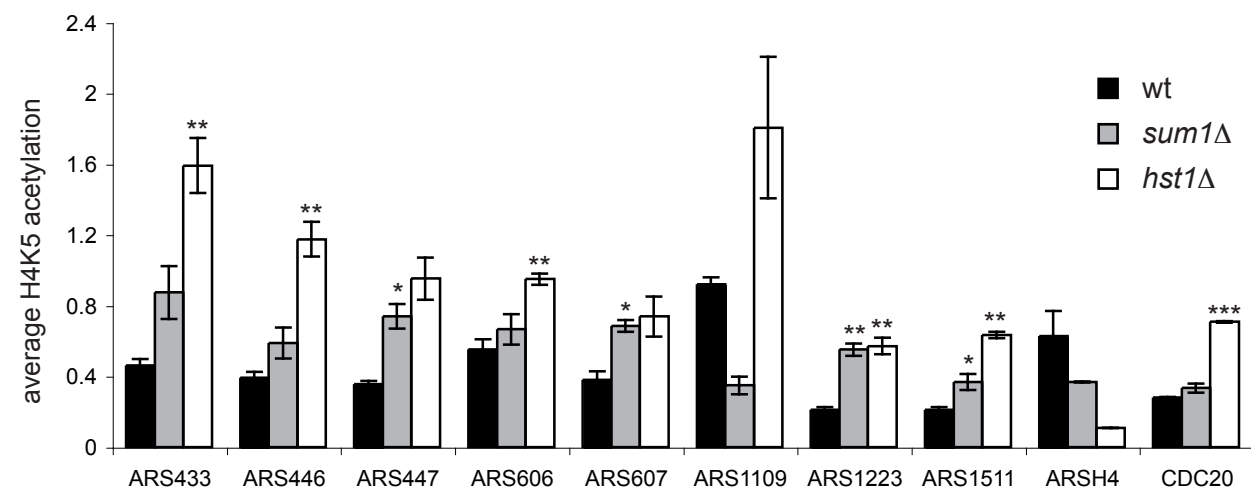

**B**

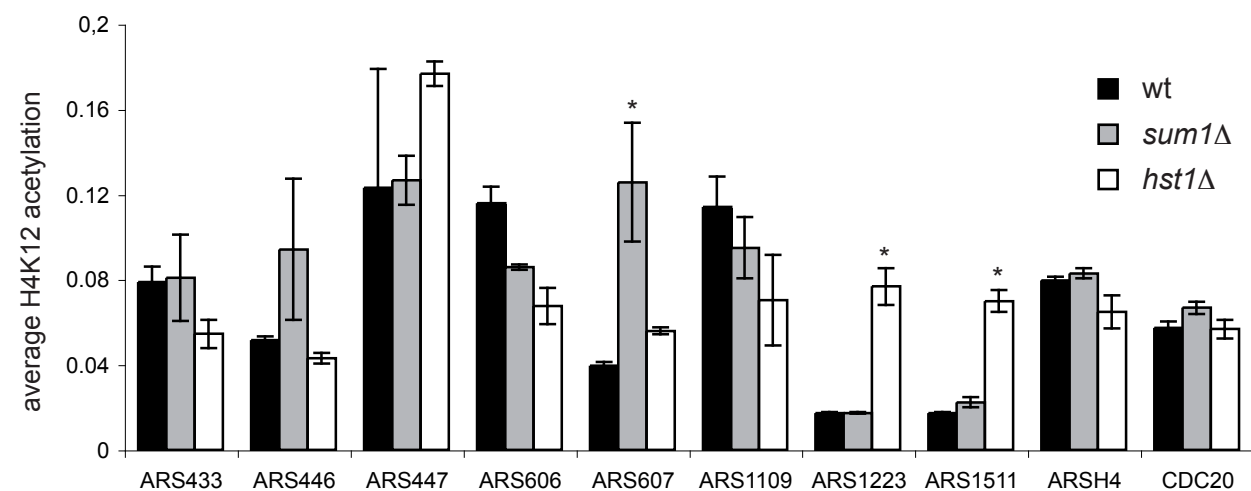

**C**

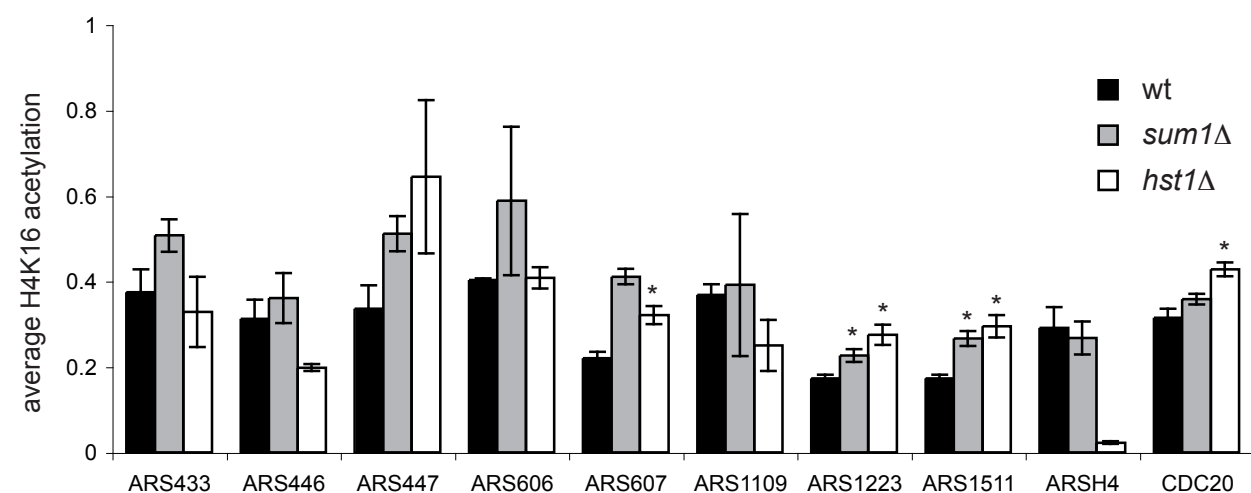

**D**

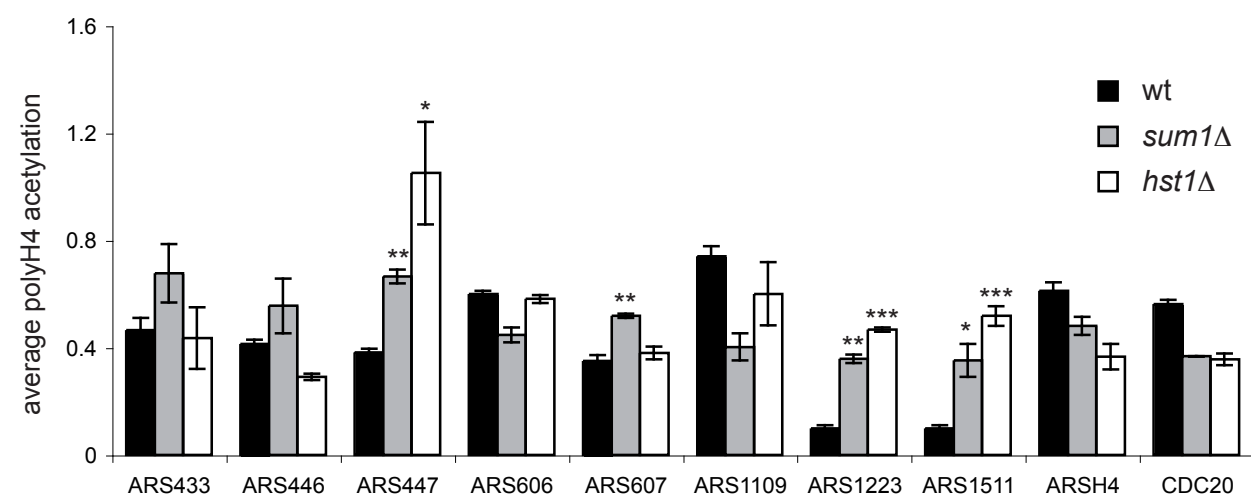

# Experiment II

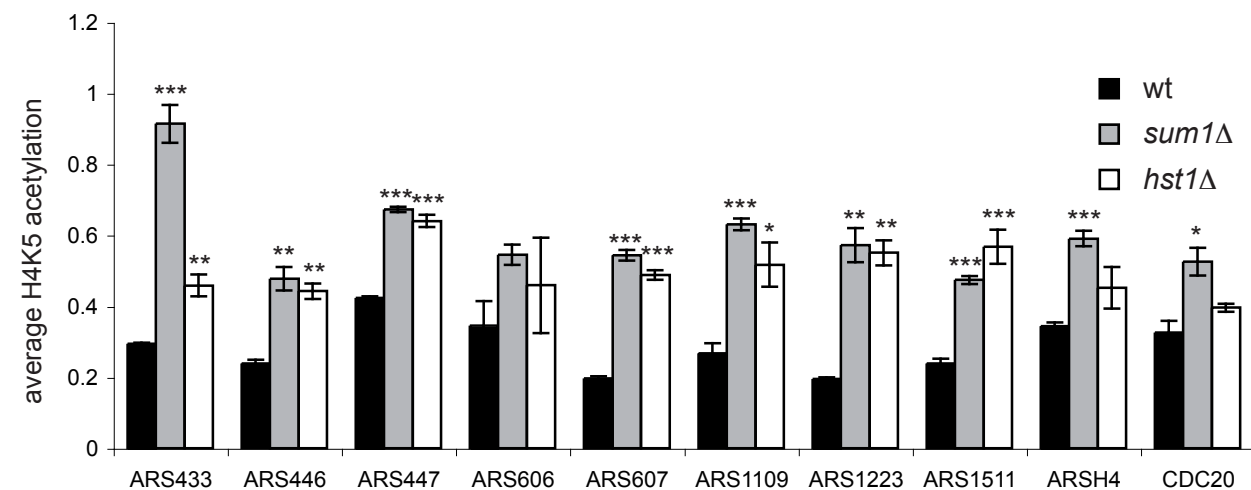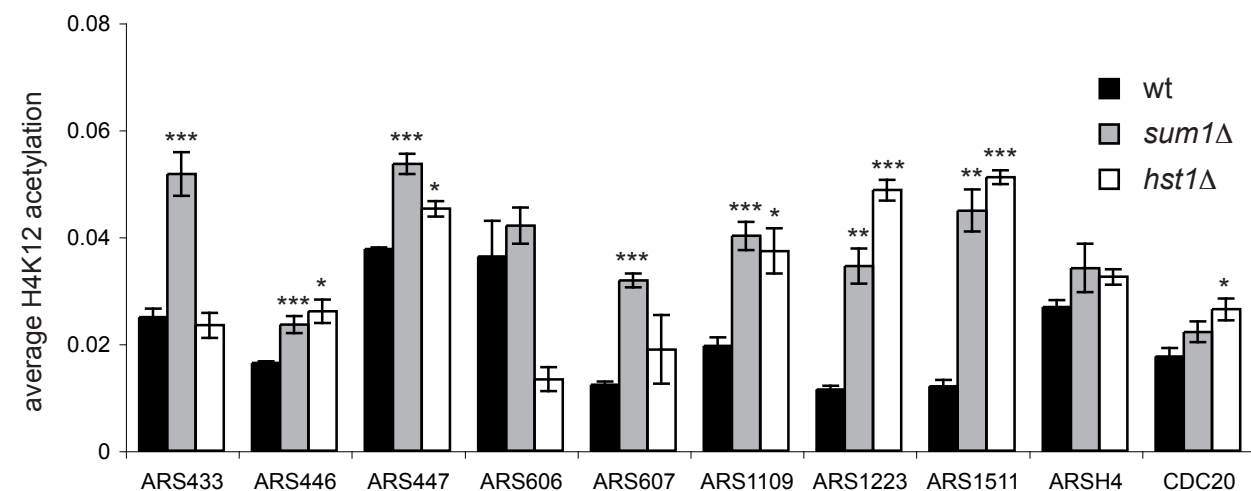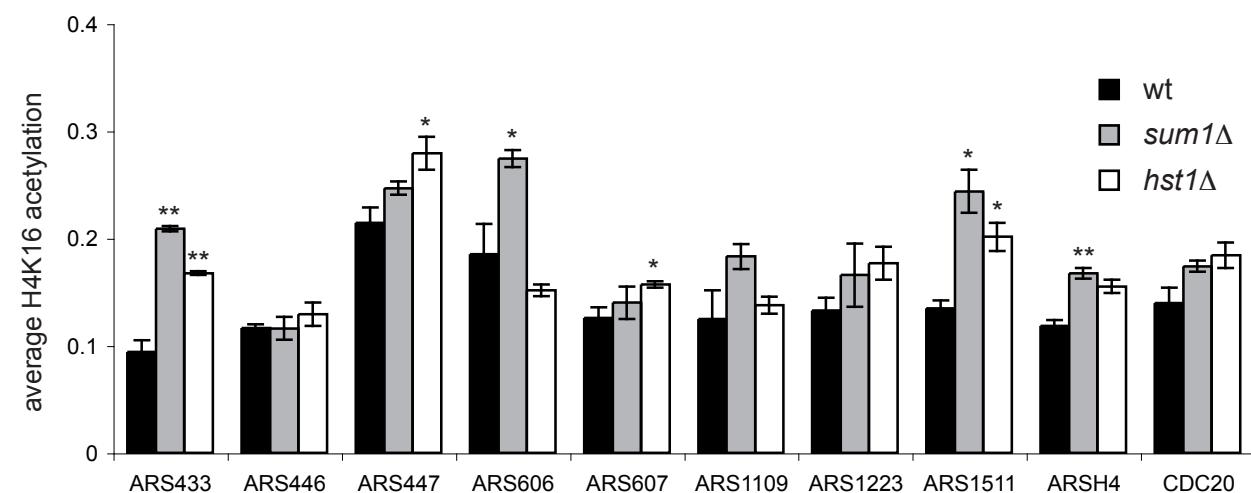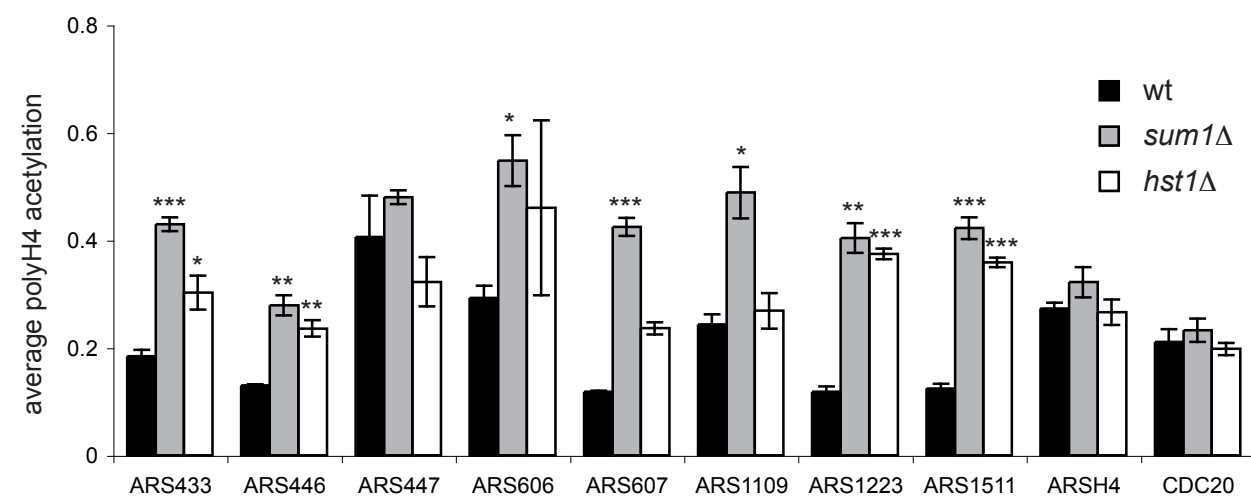

Supplement: Additional file 2 — Significance levels for increased H4 acetylation in sum1Δ and hst1Δ strains. The amount of DNA from immunoprecipitated wild-type (wt, AEY2), sum1Δ (AEY3358) and hst1Δ (AEY1499) strains with anti-acetyl-histone H4 antibodies is shown relative to the input DNA. Quantitative real-time PCR was performed for eight selected origins and two controls (ARSH4 and CDC20). Diagrams show the results for the two independent experiments. Error bars represent the average of three samples each. * (P < 0.05), ** (P < 0.01) and *** (P < 0.001) indicate statistically significant changes (student's t test). A- anti-acetyl-histone H4 K5, B- anti-acetyl-histone H4 K12, C- anti-acetyl-histone H4 K16, D- anti-acetyl-histone H4. [file 1471-2199-9-100-S2.pdf]
